# Systematic review on the conservation genetics of African savannah elephants

**DOI:** 10.7717/peerj.2567

**Published:** 2016-10-19

**Authors:** Daniel Zacarias, Luis Mauricio Bini, Rafael Loyola

**Affiliations:** 1 Programa de Pós-graduação em Ecologia e Evolução, Universidade Federal de Goiás, Goiania, Goias, Brazil; 2 Programa de Pós-graduação Ciência para o Desenvolvimento (PGCD), Instituto Gulbenkian de Ciências, Lisboa, Portugal; 3 Departamento de Ecologia, Universidade Federal de Goiás, Goiania, Goias, Brazil

**Keywords:** Molecular ecology, Ex-situ conservation, Research trends, Threatened species, Conservation assessment

## Abstract

**Background:**

In this paper we review the conservation genetics of African savannah elephants, aiming to understand the spatio-temporal research trends and their underlying factors. As such, we explore three questions associated to the conservation genetics and molecular ecology of these elephants: (1) what are the research trends concerning the conservation genetics of *Loxodonta africana*? (2) Do richer countries conduct more research on the genetics of African elephants? (3) Which attributes influence where scholars conduct their research?

**Materials and Methods:**

We examined available peer-reviewed publications from 1993 to 2014 in complementary online databases, including the ISI/Web of Science (WoS), Scopus and Google Scholar (GS), and searched for publications in scientific journals as well as in the reference section of these publications. We analyzed the annual trend of publications in this field of research, including the number of authors, levels of collaboration among authors, year of publication, publishing journal and the countries from where genetic samples were collected. Additionally, we identified main research clusters, authors, and institutional collaborations, based on co-citation and co-occurrence networks.

**Results:**

We found that during the study period there was a positive trend in the number of publications and a reduction in the number of authors per paper. Twenty-five countries contributed, with the majority of publications authored by researchers in the USA, Kenya and South Africa. The majority of samples were collected in Kenya, Tanzania and South Africa. Research outputs are associated with the existence of long-term conservation/research projects and research potential as measured by the literacy rate and the number of higher education institutions in a country. Five research clusters were identified, focusing on the origin and evolution of the species, methodological issues and the relatedness among elephant species.

**Conclusions:**

Research in this field should be expanded to additional countries harboring elephant populations to enable a more comprehensive understanding of the population structure and genetic differentiation of the species, and to cope with challenges associated with the conservation of the species such as illegal hunting, habitat fragmentation, species reintroduction and climate change.

## Introduction

Elephants have been present in the African savannah for a long period of time, influencing ecological processes and facilitating herbivory (Guldemond & Van Aarde, 2008; Graham et al., 2009; Campos-Arceiz & Blake, 2011). However, in recent times this species has been extirpated from large portions of its habitat, mainly as a result of habitat fragmentation and isolation, poaching for ivory, human-elephant conflicts and a small amount of hunting for meat consumption (Douglas-Hamilton, 1987; Blanc et al., 2005; Ntumi, Ferreira & van Aarde, 2009; Dunham, 2012; Wittemyer, Daballen & Douglas-Hamilton, 2013; Booth & Dunham, 2014). Extensive research has been undertaken to understand this species from a range of perspectives and to support better management strategies; among these, the genetic perspective has been gaining interest (Roca & O’Brien, 2005; Archie & Chiyo, 2012; Roca et al., 2015).

Due to the increasing need to improve elephant management strategies from a genetic perspective it is essential to identify trends and gaps in the literature to direct research efforts and support policy making (Roca et al., 2015). As such, illuminating the pathways of research by illustrating research trends, level of collaboration between authors and institutions and the most focused sub-topics are crucial tasks. This may improve our understanding of what has been and is currently being done and the major research gaps (Roy & Goswami, 2013; Shahram et al., 2013; Borrett, Moody & Edelmann, 2014).

To achieve this task, in this paper we conducted a systematic review of existing literature on the application of genetics to the conservation of African savannah elephants, to understand the temporal and spatial patterns of research, the degree of authors’ and institutions’ collaboration and the geographical distribution of sampling efforts. In particular, we answer three questions: (1) What are the current trends in research on the conservation genetics of *Loxodonta africana*, considering the number and year of publication, number of authors, main research line, origin of samples and country of publication? (2) What is the spatial distribution of research (samples and outputs) associated with the conservation genetics of African savannah elephants? Do richer countries conduct more research on the conservation genetics of African elephants? (3) Which attributes influence where scholars conduct their research? Answers to these questions are important to direct research efforts that are relevant to the conservation of African savannah elephants. For instance, understanding which elephant populations are more studied can direct efforts to less-studied populations and result in an improved understanding of the genetic variability across the entire range.

## Materials and Methods

### Literature search, criteria for inclusion and characterization

To develop this study we systematically reviewed available publications related to the conservation genetics and molecular ecology of African savannah elephants. Publications were primarily searched using the ISI Web of Science (WoS) platform (http://webofknowledge.com), Google Scholar (GS; http://scholar.google.com) and Scopus (http://scopus.com) using the search terms “Elephants” NOT “*Loxodonta cyclotis*” NOT “*Elephas maximus*” AND “Genetics” AND “Molecular Ecology,” for the period 1945–2014.

To increase the coverage of the available literature, we also surveyed specialized journals (Table S1) and applied a snowball technique in which the bibliographic section of available publications was used as a source for the identification of new papers (Almeida-Filho et al., 2003). For each publication, we retrieved information including the author name(s), institution and country, publication title, year of publication, journal title, the type of publication (papers, theses, reviews, book chapters), sample types used (tissue, feces, ivory) and the country where the sample was collected. Based on the title, abstract and methodology, each publication was assigned to one of the research lines listed in Table 1.

**Table 1:** Description of the research lines adopted in this study.

	Type	Description
1	Research line 1 (RL1)	Management and reintroduction of captive populations and restoration of biological communities
2	Research line 2 (RL2)	Description and identification of individuals, genetic population structure, kin relationships and taxonomic relationships
3	Research line 3 (RL3)	Detection and prediction of the effects of habitat loss, fragmentation and isolation on populations
4	Research line 4 (RL4)	Detection and prediction of the effects of hybridization and introgression
5	Research line 5 (RL5)	Understanding the relationships between adaptation or fitness and genetic characters of individuals or populations
6	Research line 6 (RL6)	DNA forensics

**Source:**

Adapted from Allendorf, Luikart & Aitken (2013).

### Analysis of publication metrics

To assess the trends in the research associated with the conservation genetics and molecular ecology of African savannah elephants, we measured three indicators: the growth rate of publications (Rp), the doubling time of publications (Dt) and the degree of collaboration (DC). These indicators have been suggested as a framework to understand how a specific field of research grows temporally and the extent to which authors participate in group research (Larsen & von Ins, 2010; Pautasso, 2012; Bornmann & Mutz, 2015; Saravanan & Dominic 2014). Details of calculations are presented in Supplemental Information S1.

Apart from these metrics, we attempted to identify major research clusters (e.g., text corpus or author and institutional collaboration). This task was performed using VOSviewer (van Eck & Waltman, 2010), a software designed to visualize research landscapes and ascertain degrees of collaboration between authors and organizations (Heersmink et al., 2011; Waltman & van Eck, 2013; van Raan, 2014; van Eck & Waltman, 2010). Although very recently developed, this software has been extensively used due to its ability to map networks of publications, authors or journals by means of co-citation and co-occurrence networks (Waltman, van Eck & Noyons, 2010; van Eck & Waltman, 2014; Rodrigues et al., 2014; see also Borrett, Moody & Edelmann, 2014).

### Geographic distribution of samples and research outputs

To illustrate the spatial distribution of samples and research outputs, we created a database specifying the country where samples were collected, the respective number of publications based on these samples and the geographical origin of all authors of each publication. Further, we mapped the distribution of samples using ArcGIS 10.3 (see also Yaoyang & Boeing, 2013; Fu et al., 2014; Bundschuh et al., 2012). To avoid overestimating the number of participating institutions and authors’ countries of origin, we used the following approach: if an author institution was repeated in a single publication, it was considered as a single appearance; if a single author belonged, to two or more institutions at the time of publication, all were considered as participating and added to the database (Pan, Kaski & Fortunato, 2012).

We used stepwise multiple linear regression to identify the factors that influence research outputs, measured by the total number of papers associated with each country. For this, we used the number of higher education institutions in the country, the country literacy rate, Gross Domestic Product per capita at the purchasing power parity (GDP-PPP), the percentage of GDP directed to research and development expenditures, the environmental performance index, the number of elephants and associated range in the country and presence or absence of long-term conservation and/or research projects in each country. Details on the variables are presented in Table S3. All calculations were done using the Statistical Package for the Social Sciences (SPSS) v.22.

## Results

**Figure 1:**
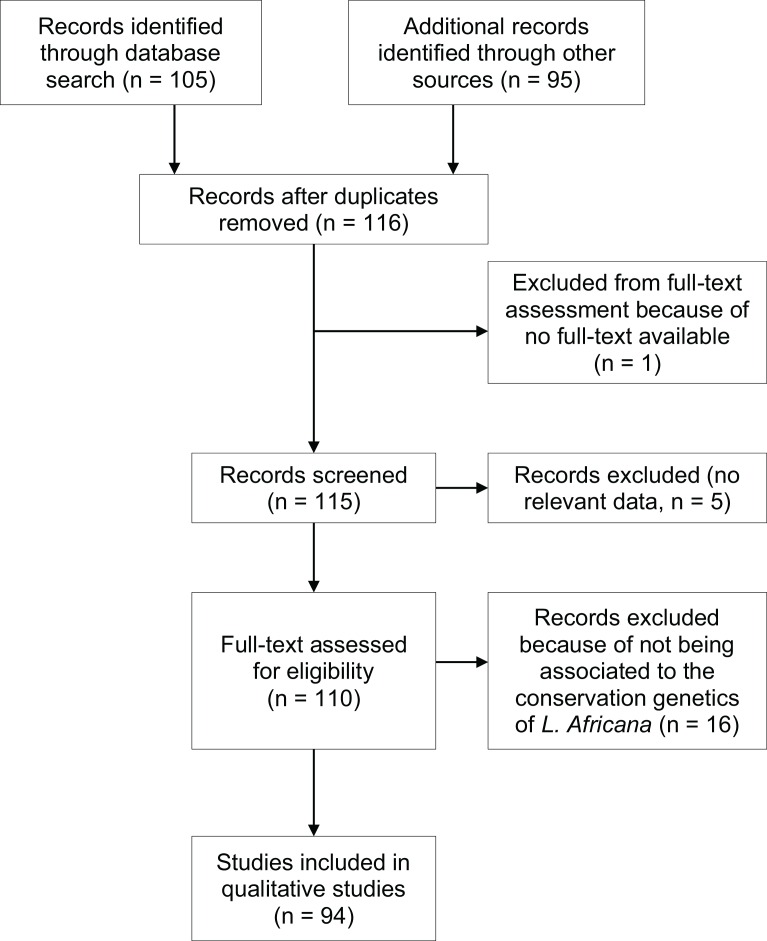
PRISMA flow diagram (adapted from Moher et al., 2009).

Ninety-four publications were found in this study (Fig. 1). Although the intention was to retrieve publications from 1945 to 2014, we were only able to collect papers published from 1993 onward (Table S1).

Most of the publications were peer-reviewed journal articles (n = 85, 90.4%); we also reviewed theses (n = 6, 6.4%), reports (n = 2, 2.1%) and one book. Of these, journal articles were subdivided into 71 research articles, 10 research notes and four reviews. These were subdivided into master (n = 3) and doctoral (n = 3) and one publication was documented as a chapter in an edited book. These documents were published in 55 journals, books, or as theses, with Molecular Ecology (n = 10, 10.64%), Heredity (n = 6, 6.38%) and Conservation Biology (n = 5, 5.32%) being the most targeted journals (Table 2).

**Table 2:** Main journals and respective number of publications on conservation genetic of elephants from 1993 to 2014. Only journals with a minimum of two papers are presented (n = 18 out of 55).

	Repository	Frequency (n out of 94)	Ratio (%)
1	Molecular Ecology	10	10.64
2	Heredity	6	6.38
3	Conservation Biology	5	5.32
4	PLoS ONE	4	4.26
5	Animal Behaviour	3	3.19
6	Animal Conservation	3	3.19
7	Conservation Genetics Resources	3	3.19
8	Molecular Ecology Notes	3	3.19
9	African Journal of Ecology	2	2.13
10	BMC Evolutionary Biology	2	2.13
11	Conservation Genetics	2	2.13
12	Journal of Zoology	2	2.13
13	Molecular Ecology Resources	2	2.13
14	PNAS	2	2.13
15	Proceedings of the Royal Society of Biology	2	2.13
16	Science	2	2.13
17	University of Pretoria	2	2.13
18	The Royal Society Academy of Sciences	2	2.13

**Figure 2:**
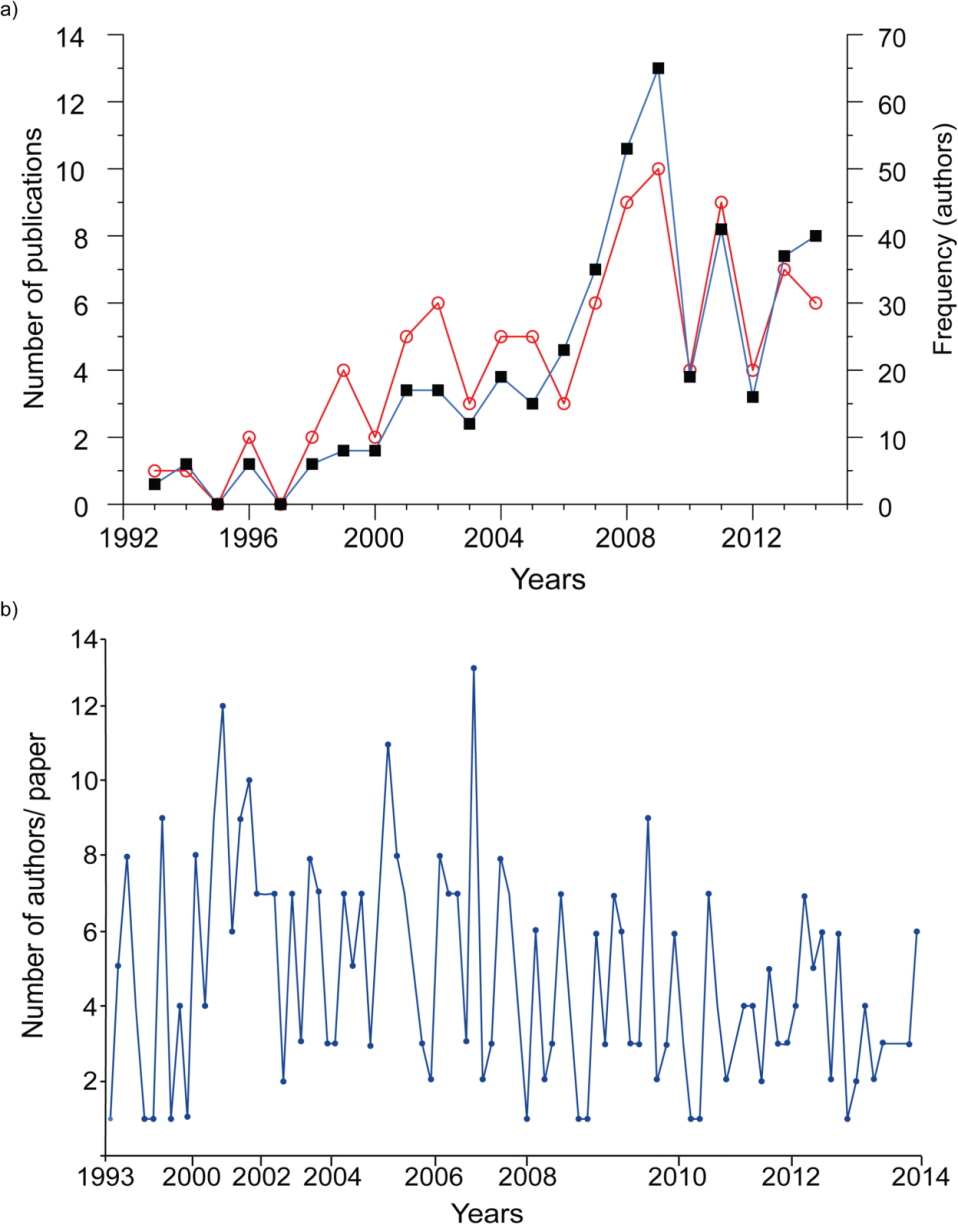
Dynamics of research productivity. (A) Temporal distribution of publications and authors associated to the conservation genetics of *Loxodonta Africana*. The red line represents the number of authors/year and the blue line, the number of publications. (B) Time series analysis of the number of publications per paper during the study period.

Documents were published yearly from 1993 to 2014, with the exceptions of 1995 and 1997 for which no publication were registered (Fig. 2A), written by a total of 246 individual authors. The year 2010 was the most productive, with 10 publications written by 65 authors. Over the years, the number of published papers increased (mean = 4.7, SD = 2.64), as well as the number of authors/year (mean = 22.3, SD = 17.26). However, the number of authors/paper (i.e. collaboration) did not increase (r = 0.277, p > 0.05, Fig. 2B), as indicated by the large number of papers authored by less than fewer that five authors (mean = 4.74, SD = 2.76).

Publications were based on the three primary types of samples. Some publications were based on a single type of sample (feces, tissue or ivory) and others considered a combination of samples (tissue and blood, tissue and feces). For example, 31.9% of the publications (n = 30) were written based on fecal samples, 18.1% (n = 17) were based on tissue samples and 10.6% (n = 10) combined fecal and tissue samples. Nine documents (9.57%) were based on ivory samples.

Most research focused on the description and identification of individuals, genetic structure, kinship and taxonomic relations (n = 33, 35.87%); and on understanding the relationship between adaptation, fitness and the genetic traits of individuals and populations (n = 25, 27.17%). The period from 2008 to 2012 had the largest number of publications in any five-year period (n = 36), most of them focusing on the description and identification of individuals, the genetic population structure, kin relationships and taxonomic relations (RL2, n = 19); and the detection and prediction of the effects of habitat loss, fragmentation and isolation on populations (RL3, n = 9).

**Figure 3:**
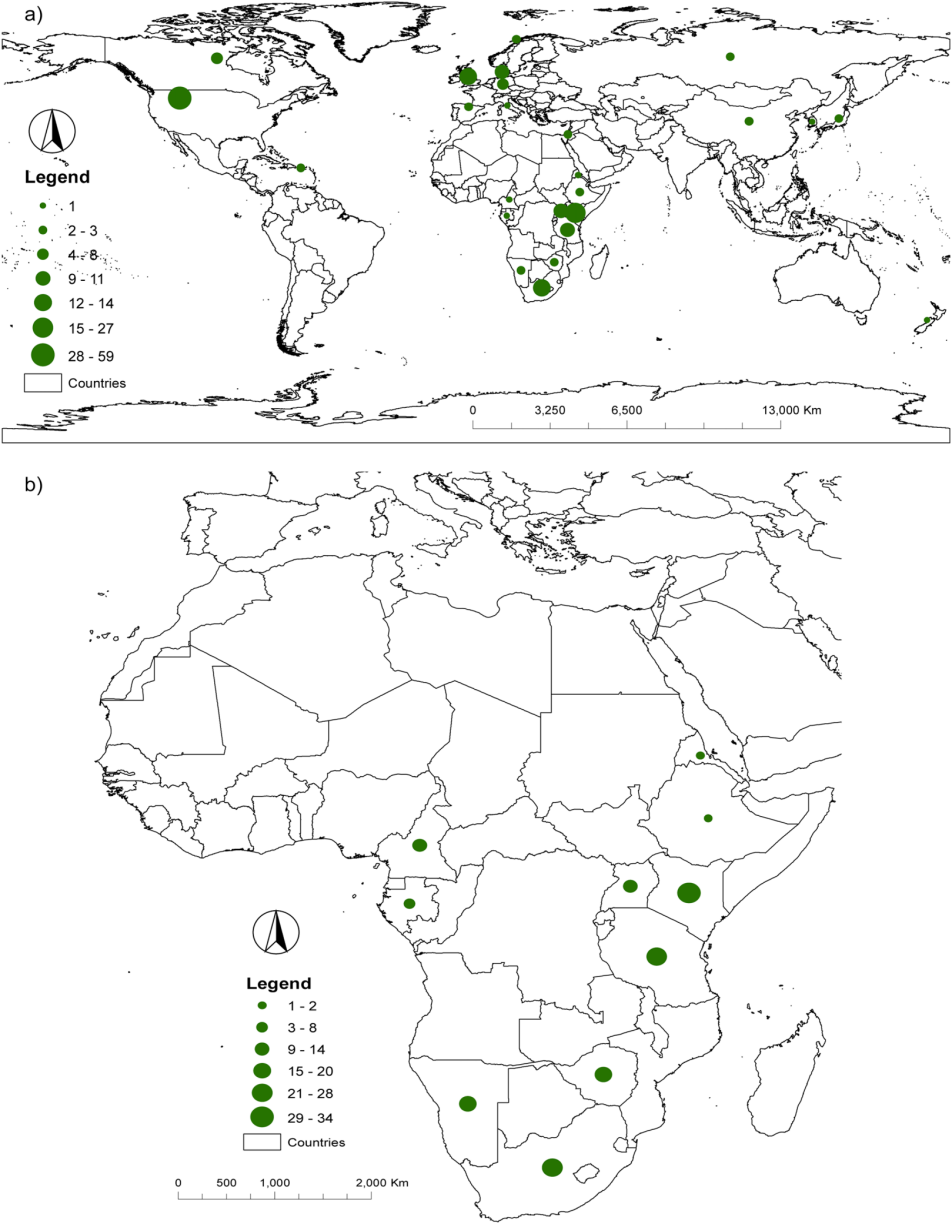
Geographic distribution of (A) authors and (B) samples collected (in publications).

Most of the research on the genetics of savannah elephants was conducted by researchers based at institutions in the North American continent (n = 162, 46.55%) followed by researchers based in Africa (n = 99, 28.45%) and researchers in Europe (n = 59, 16.95%). Despite having the larger number of researchers, the North American continent only had three participating countries (USA with 150 author credits, Canada with 11 and Puerto Rico with one). Africa held the largest number of participating countries (n = 10) and was the second most represented continent with extensive participation from Kenya and South Africa (35 and 23 author credits, respectively). A great majority of samples were collected in range countries in Africa, with very few collected from captive elephants outside Africa, whose origin were known or could be assigned to a range country in Africa. Most samples were collected in Kenya (34 publications), Tanzania (28 publications) and South Africa (27 publications) (Fig. 3).

We used multiple regression to determine the factors that could predict research outputs per country and the choice of a country for sampling. The number of papers per country was influenced by the presence of long-term projects (b = 0.74, t(18) = 4.54, p < 0.001), the literacy rate of the country (b = 0.478, t(18) = 3.54, p < 0.01) and the number of higher education institutions (b = 0.325, t(18) = 3.23, p < 0.01). The combination of these factors explained the variance in the number of papers per country by 85% (F(3,15) = 28.430, p < 0.001, R^2^ = 0.85, R_adjusted_^2^ = 0.821).

**Figure 4:**
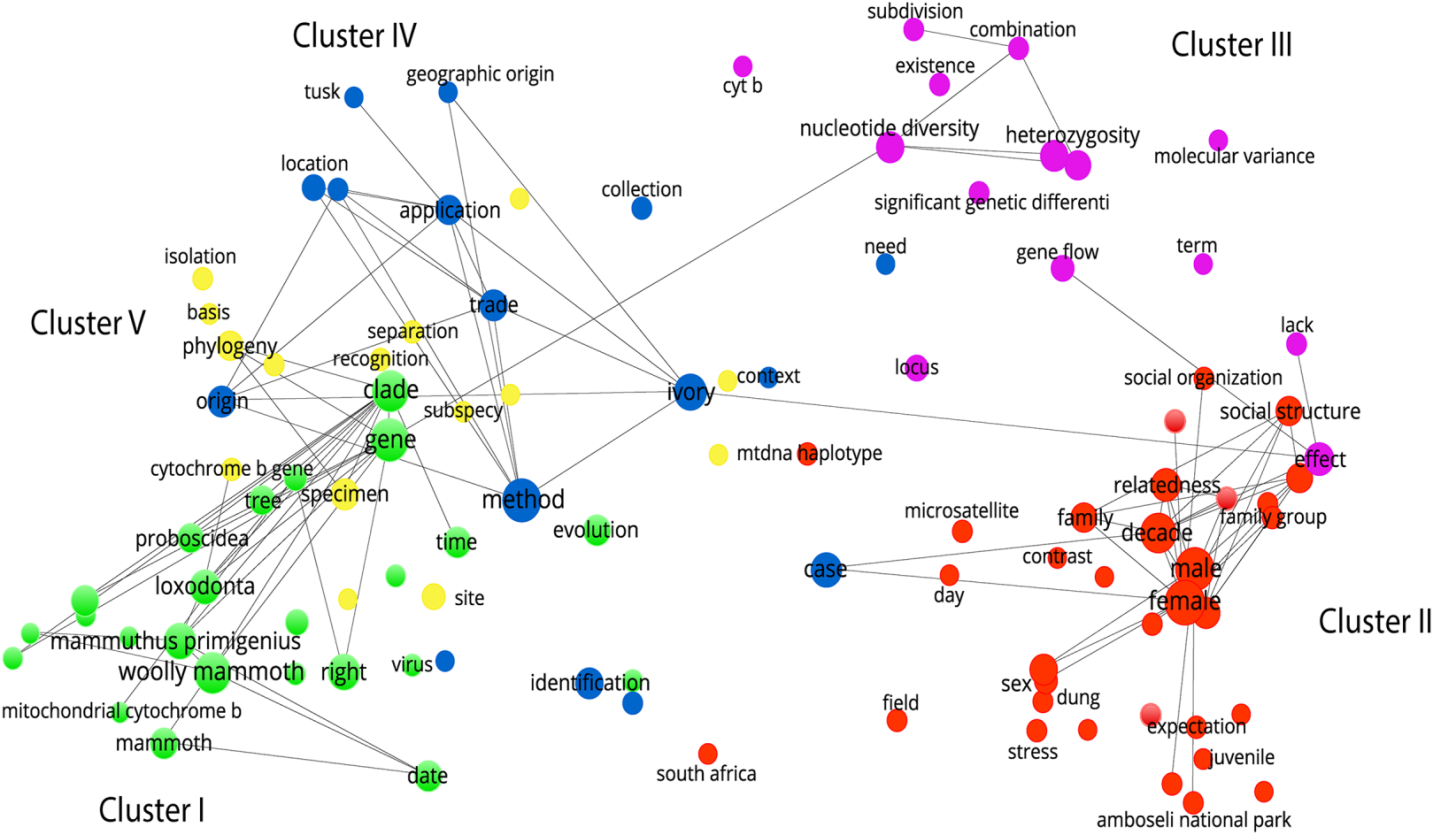
Network analysis of research clusters based on text corpus. Cluster I: phylogenetic relations and species’ evolution. Cluster II: species relatedness and group structure. Cluster III: gene flow and genetic differentiation. Cluster IV: utilization of DNA tools to track the geographic origin of ivory. Cluster V: species relatedness from a genetic perspective.

The publication of research on the molecular ecology of African elephants had an average (5-year period) growth rate of 0.79, an average doubling time of 1.82 and a collaborative coefficient of 0.676 (Supplemental Information). The growth rate was higher in the period 1998–2002 (Rp = 1.75) and lower in 2013–2014 (Rp = 0.15), and the doubling time was higher in the period 2013–2014 (Dt = 4.66) and lower during 1998–2002 (Dt = 0.40). As previously indicated, there were a total of 246 individual authors published during the timeframe, most of them with single author credits (n = 181, 73.58%). Twenty-five authors were responsible for 39.37% (n = 176) of all authorships. A network analysis of the DC based on bibliographic coupling of authors revealed five major research clusters (Fig. 4)—namely, phylogenetic relationships and species evolution (Cluster I), relatedness of species and group structure (Cluster II), gene flow and genetic differentiation (Cluster III), use of DNA tools to track the geographic origin of ivory (Cluster IV); and individual and population relatedness from a genetic perspective (Cluster V).

**Figure 5:**
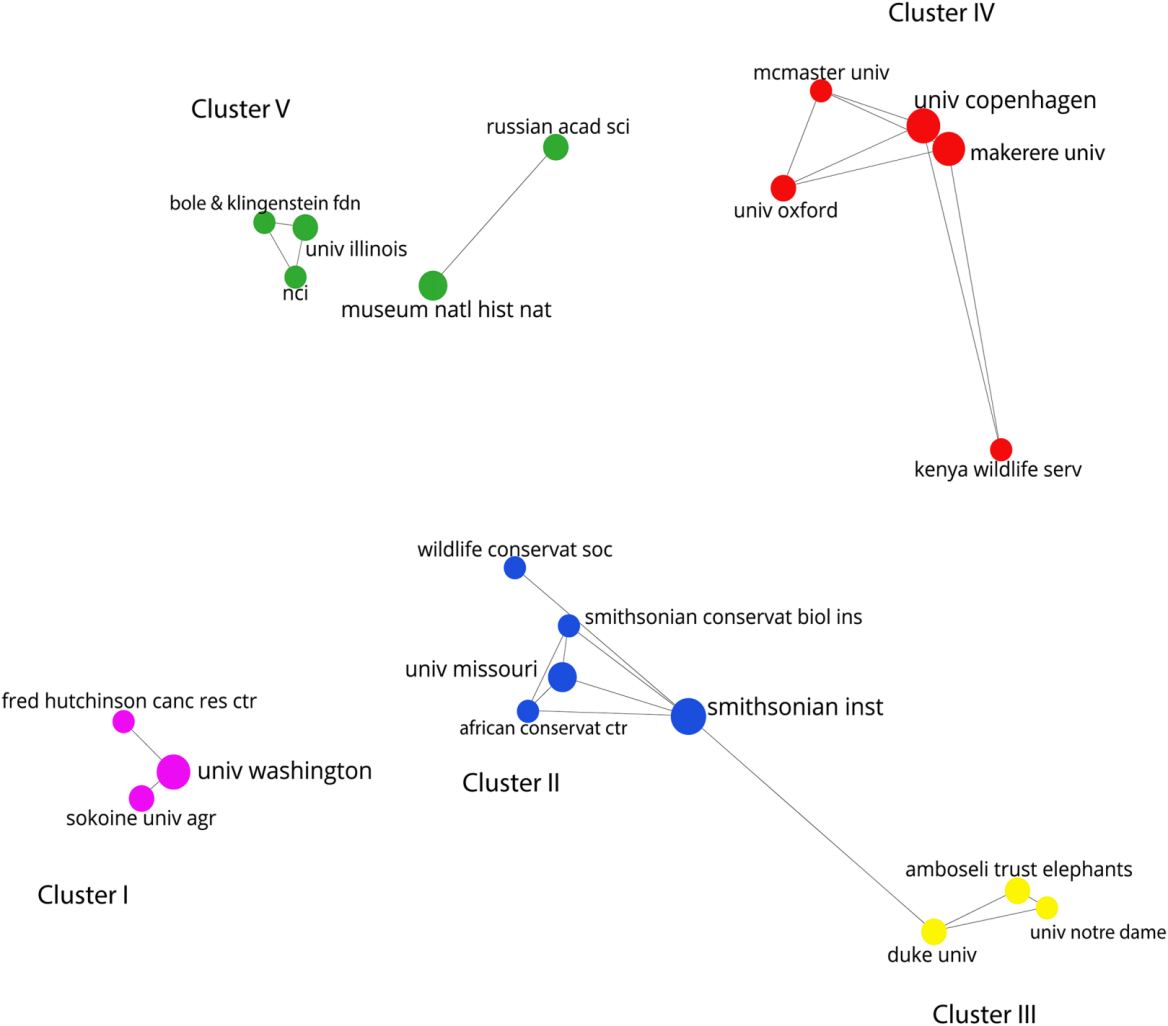
Collaborative network between research institutions. Larger nodes indicate the leading institution in each cluster.

Based on the DC between institutions, the network showed that the Smithsonian Institute, the University of Washington (USA) and the Copenhagen Institute (Denmark) dominate the research on the conservation genetics of African savannah elephant, while the Makerere University is the main institution in Africa. These institutions are involved in a research network that spans the five research clusters, headed by the University of Washington (Cluster 1), the Smithsonian Institute (Cluster 2), Duke University and the Amboseli Trust for Elephants (Cluster 3), the University of Copenhagen (Cluster 4) and the National Museum of Natural History of the United States (Cluster 5) (Fig. 5).

## Discussion

Several important issues associated with the conservation genetics and molecular ecology research about African savannah elephants can be identified from this study. First, there was a positive trend in the number of publications per year and a reduction in the number of authors per paper during the study period. Second, Kenya, Tanzania and South Africa dominate the list of countries from which samples were collected, while the USA, Kenya and South Africa head the list of countries from which the most studies were published. Finally, we showed that research outputs are influenced by the presence of long-term research projects in a country and by the research potential, proxied by the literacy rate and the number of tertiary institutions per country.

Despite the fact that research on the conservation genetics of African elephants attracts researchers from different countries and represents a growing trend, when compared to studies of other species its growth rate remains weak—especially considering that elephants are iconic species threatened by high levels of poaching and habitat fragmentation. However, the increasing interest in studies on the conservation genetics of African elephants illustrated in this paper is not an isolated phenomenon. Interest in other species and groups of organisms has also grown, especially following the publication of the first conservation genetics’ papers in the 1970s (see Allendorf, Luikart & Aitken, 2013; Hedrick, 2001). For example, since then, there has been observed growing trends in 16 sub-specialties of genetics, including conservation genetics (Sangam et al., 2014); plant genetics and breeding in several countries, including Brazil, China, India, UK and USA (Pan, Kaski & Fortunato, 2012; Garg et al., 2011); plants of the Brazilian tropical savannah known as the Cerrado (de Souza & Santos, 2014; Diniz-Filho et al., 2012; Schlottfeldt et al., 2015a; Schlottfeldt et al., 2015b); and amphibians (Emel & Storfer, 2012). Such increased interest is associated with advances in molecular biology techniques (de Souza & Santos, 2014). However, considering the ecological role of African savannah elephants and their threatened status, the number of papers published in this field is still very low and more research is needed. Compared with other iconic species, the conservation genetics of African elephants was the focus of a lower number of studies (n = 94) than similar studies on bears (n = 458), deer (198 studies), whales (n = 138 studies), sharks (n = 108) and turtles (n = 254).

The need to understand the potential effects of human-induced activities such as habitat fragmentation and poaching for ivory may be fueling interest in researching their conservation from a genetic perspective. This assumption is supported by the temporal trend in the number of publications by research line. Results indicate that the most studied research lines are those associated with the relationship between adaptation, fitness and individual characteristics (see Fig. 2), illustrating the growing concern over the future adaptability of the species. On the other hand, no study has yet attempted to associate the genetic variability of the African elephant population with climate change.

As in other research fields (Brito, 2008; Carneiro, Nabout & Bini, 2008; Lolis et al., 2009; Sarragiotto & Benedito, 2013; Morais & Cianciaruso, 2014), researchers from the USA or associated with USA-based organizations dominate the conservation genetics of African elephants. Although this is positive, it would be more interesting and cost-efficient if more African researchers could be involved in this field, thus enabling long-term African research and capacity building. From 1993 to 2014, 348 institutions were involved in conservation research on African elephants, nearly half of which (n = 150) were based on the USA. Of these, the University of Washington dominates with 24 associated researchers, followed by the University of Illinois. With respect to range countries, the Makerere University in Uganda is the leader with 15 researchers.

Although non-range countries do the most research, there is a high degree of cooperation among researchers and institutions. Researchers associated with the University of Washington (USA) worked more closely with their counterparts at the Fred Hutchinson Cancer Research Center (USA) and the Sokoine University (Tanzania), while researchers from University of Copenhagen (Denmark) tended to be associated with researchers from the Makerere University and the Kenya Wildlife Service. Interestingly, researchers from Kenya, Uganda and Tanzania appeared to have strong collaboration levels, appearing in co-authorship with researchers that dominate the field, but rarely collaborating among themselves. This contradicts the general idea that regions close to each other are more inclined to collaborate (Hoekman, Frenken & van Oort, 2009) and supports the idea that social proximity is essential to achieving close collaboration (Ponds, van Oort & Frenken, 2007; Hoekman, Frenken & van Oort, 2009; Hoekman, Frenken & Tijssen, 2010; Ben Letaifa & Rabeau, 2013; Marrocu, Paci & Usai, 2013).

Neither the number of elephants nor the range size appeared to directly influence researchers’ decision to collect samples in a country, but the presence of long-term conservation/research projects and the research potential may be important factors. This can be evidenced by the fact that Kenya, Tanzania and South Africa are the most sampled countries, as they have well-established conservation projects such as the Amboseli Elephant Research Project (operating in Kenya since 1976 in a collaborative network), the David Sheldrick Wildlife Trust (also in Kenya since 1977), the Tarangire Elephant Project (established in 1993 in Tanzania) and the long-running wildlife conservation programs developed by South African National Park authorities through the Kruger National Park, the Knysha Elephant Park and the Addo Elephant National Park. Although not directly associated with the choice of sampling, the number of elephants and the range size explained 30.6% of the presence of long-term research projects, with the number of elephants being the predictor of the presence of long-term research projects. Considering these results, it can be assumed that the number of elephants in a country potentially attracts the implementation of research projects, which in turn increase the number of samples that are collected.

Results of this study also indicate that most range areas have been studied, although there are still countries with large elephant populations, but few samples. For example, it appears that East African populations are the most researched in country-wide and inter-country studies, while the remaining regional populations have only scattered studies that may not provide reliable information for regional management. For example, while the molecular biology of populations in Uganda, Kenya and Tanzania are well documented from single-country populations and between countries, these studies are scarce in other regions, which can challenge conservation efforts.

## Conclusions

In this paper, we answered three main questions associated with the molecular ecology and conservation genetics of African savannah elephants. Our results show that the conservation genetics of African savannah elephants is attracting an increasing number of researchers, with particular focus on the description and identification of individuals, genetic population structures, kin relationships and taxonomic relationships. Among the common research lines, the application of DNA to track the geographic distribution of ivory is gaining importance.

Researchers based in the USA, Denmark, Canada and Kenya conducted most of the research, using samples primarily collected in Kenya, South Africa and Tanzania. This trend indicates that genetic research on African elephants does not cover all known range countries and that significant populations are still genetically unknown. This might have implications for processes of restoring populations, based on the assumption that translocation programs with genetically different populations may not be successful. Efforts are needed to address this issue and incorporate other range countries into these studies.

## Supplemental Information

10.7717/peerj.2567/supp-1 Supplemental Information 1 Supplementary Methods.

10.7717/peerj.2567/supp-2 Supplemental Information 2 Summary of the studies associated to the conservation genetics and molecular ecology of the African savannah elephants (*Loxodonta africana*).

10.7717/peerj.2567/supp-3 Supplemental Information 3 The PRISMA checklist.

10.7717/peerj.2567/supp-4 Supplemental Information 4 Indicators used to evaluate the factors that influence the decision to sample in a country.

## Additional Information and Declarations

### Data Deposition

The following information was supplied regarding data availability: The raw data has been supplied as Supplemental Dataset Files.
