# Supplementary material for: Systematic review on the conservation genetics of African savannah elephants
Source: PeerJ. 2016 Oct 19;4:e2567. doi: 10.7717/peerj.2567 (PMC5075695; doi:10.7717/peerj.2567)
Supplement: Supplemental Information 1 [file peerj-04-2567-s001.docx]

**Supplementary Information**

**Supplementary Methods S1**. Research trends and degree of collaboration between authors

To assess the trends in the research associated to the conservation genetics and molecular ecology of African savannah elephants (*Loxodonta africana*), we used a combined approach in which temporal trends were assessed in terms of the number of papers published over a 5 period time lapse and the thematic trend was assessed in terms of the distribution of all publications into thematic lines. Additionally, research outputs were evaluated in terms of the relative growth and doubling time of publications. The relative growth rate of literature (RGR) is defined as the increase in the number of publications per unit of time (Mahapatra, 1985), mathematically represented by Equation 1, whilst the relative Doubling Time of Publications (DT) is the time required for the article to become double of the existing amount (Sangam & Keshava 2003), mathematical calculated by Equation 2.

$R\left( P \right)= \frac{{Log}_{e}2P- {Log}_{e}1P}{2^{T}- 1^{T}}$ (1)

Where:

R(P) = relative growth rate of publications over a specific period of time

Log_e_1P = Log of initial number of articles

Loge2P = Log of final number of articles

2^T^ – 1^T^ = The unit difference between the initial time and final times

$DT= \frac{{Log}_{e}2}{R(P)}=\frac{0.693}{R(P)}$ (2)

Where, DT is the doubling time and other variables are consistent with Equation 1.

The degree of collaboration was assessed in terms of the collaborative index (CI), degree of collaboration (DC) and the collaborative coefficient (CC), the same approach followed by (Govindaradjou & John, 2014; Saravanan & Dominic, 2014). The CI is the number of papers and is considered a measure of mean number of authors (Lawani, 1980) and is calculated using Equation 3, where Fj = number of papers having j authors, N = total number of papers and A = total number of authors.

$CI= \frac{\sum_{j=1}^{A} jfj}{N}$ (3)

The DC is the measure of the proportion of multiple authored papers and was calculated by applying Equation 4, where N*_m_* is the number of multi-authored publications and Ns is the number of single authored publications (Saravanan & Dominic, 2014).

$DC=\frac{N_{m}}{N_{m}+N_{s}}$ (4)

The CC was calculated following the approach developed by Ajiferuke et al., (1988), based on the idea that by using only the CI and the DC, although providing a fairly clear idea of the extent of collaboration in a discipline (Subramanyam, 1983), may create problems in comparative studies. Under this approach, the CC was calculated assuming that credits in publications were to be assigned as 1/n, with single authors receiving one credit, papers with two authors receiving ½ credits (half for each author) and papers with X authors received 1/x credits, such as the average credit awarded to each author of a random paper would be E[1/x), ranging from 0 to 1 (Ajiferuke et al., 1988).

References:

Subramanyam, K. (1983). Bibliometric studies of research collaboration: a review. *Journal of Information Science* 6: 33-38. doi: 10.1177/016555158300600105

Ajiferuke, I.; Burrel, Q.; Tague, J. (1988). Collaborative coefficient: a single measure of the degree of collaboration in research. *Scientometrics* 14: 421-433. Doi: 10.1007/BF02017100

Sangam, S.L & Keshava. 2003. Growth pattern of literature in the field of social science. *SRELS Journal of Information Management* 40(1): 77-84. DOI: 10.17821/srels/2003/v40i1/48888

Govindaradjou, S., & John, D. (2014). Quantitative analysis of research trends in a leading ecological journal : bibliometric study during 2003-2012. *SA Jnl Libs & Info Sci*, *80*(2011), 27–40. doi:10.7553/80-1-181

Lawani, S. M. (1980). *Quality, collaboration and citation in cancer research: a bibliometric study*. Florida State University.

Mahapatra, M. (1985). On the validity of the theory of exponential growth of scientific literature. In *The 15th IASLIC Conference Proceedings* (pp. 61–70). Banglor: IASLIC.

Saravanan, G., & Dominic, J. (2014). A Ten-year Bibliometric Analysis of Research Trends in Three Leading Ecology Journals during 2003-2012. *Journal of Information Science Theory and Practice*, *2*(3), 40–54. doi:10.1633/JISTaP.2014.2.3.4

**Supplementary Table S2**: Growth rate (Rp) and Doubling Time (Dt) of literature

| **Time (years)** | **Number of Papers** | **Cum. Papers** | $\boldsymbol{Log}_{\boldsymbol{e}}\boldsymbol{1}\boldsymbol{P}$ | $\boldsymbol{Log}_{\boldsymbol{e}}\boldsymbol{2}\boldsymbol{P}$ | **R(P)** | **Dt(P)** |
| --- | --- | --- | --- | --- | --- | --- |
| 1993-1997 | 4 | 4 |  | 1.39 |  |  |
| 1998-2002 | 19 | 23 | 1.39 | 3.14 | 1.75 | 0.40 |
| 2003-2007 | 22 | 45 | 3.14 | 3.81 | 0.67 | 1.03 |
| 2008-2012 | 36 | 81 | 3.81 | 4.39 | 0.59 | 1.18 |
| 2013-2014 | 13 | 94 | 4.39 | 4.54 | 0.15 | 4.66 |

**Supplementary Table S3**: Collaborative coefficient

| **Time (years)** | **Single** | **Two** | **Multi (3-6)** | **Mega (7-9)** | **10+** | **Total** | **CC** |
| --- | --- | --- | --- | --- | --- | --- | --- |
| 1993-1997 | 0 | 0 | 4 | 0 | 0 | 4 | 1 |
| 1998-2002 | 3 | 5 | 10 | 1 | 0 | 19 | 0.52 |
| 2003-2007 | 3 | 2 | 10 | 6 | 1 | 22 | 0.59 |
| 2008-2012 | 4 | 3 | 12 | 15 | 2 | 36 | 0.62 |
| 2013-2014 | 1 | 0 | 7 | 4 | 1 | 13 | 0.65 |
| Total | 11 | 10 | 43 | 26 | 4 | 94 | 1 |

**Supplementary Figure S1**: Co-authorship network


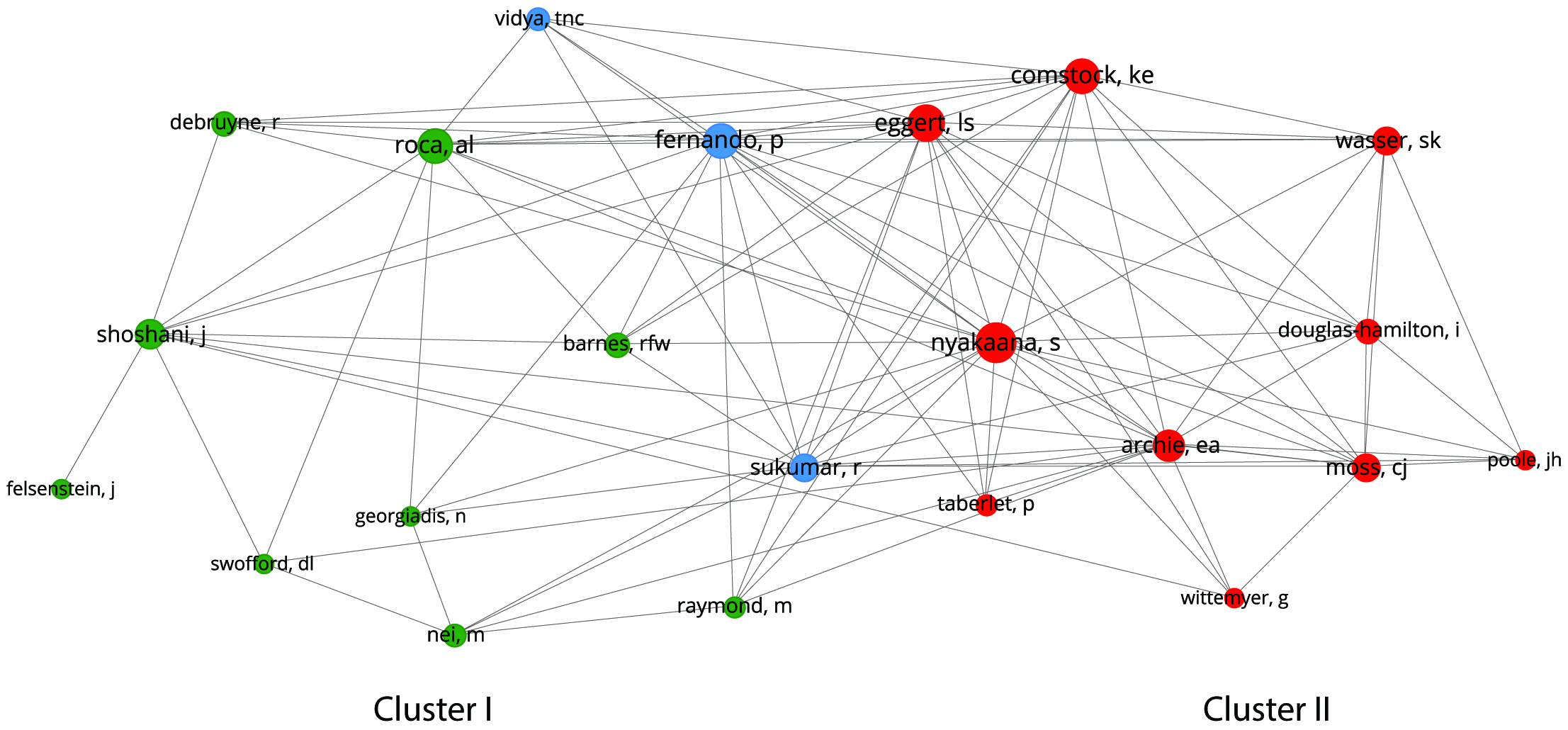


Figure S1: Co-authorship network between researchers on the conservation genetics of African savannah elephants (*Loxodonta africana*), evidencing the existence of two main groups of researchers working together and the reduced collaboration between (especially in Cluster 1). Blue nodes represent researchers that work mostly with Asian elephants but are included here as they are focused on the evolution of modern elephants from the mammoth (*Mammuthus primigenius*).
